# Lost in the scales: Alcohol-induced zinc deficiency

**DOI:** 10.1016/j.jdcr.2025.06.062

**Published:** 2025-08-22

**Authors:** Fei Ya Lai, Kevin O’Hare, Stephen Crowther, Caitriona Hackett, Kevin Molloy

**Affiliations:** a Dermatology Department, Tallaght University Hospital, Dublin, Ireland; b Department of Histopathology, Tallaght University Hospital, Dublin, Ireland

**Keywords:** acrodermatitis enteropathica, desquamation, epidermal barrier, nutritional deficiency, wound healing, zinc deficiency

A 76-year-old woman presented with a 3-week history of a well-demarcated annular scaly papulosquamous eruption, affecting her arms, legs, and buttocks, sparing the face (Fig 1, *A*,*B*). She had a background history of a long-standing clinical diagnosis of psoriasis, epilepsy, ischemic heart disease, and previous alcohol excess. Initially, the rash improved with topical corticosteroids (betamethasone valerate 0.1% w/w ointment) but flared upon discontinuation. The patient was subsequently treated with ustekinumab but did not respond to therapy.

**Fig 1:**
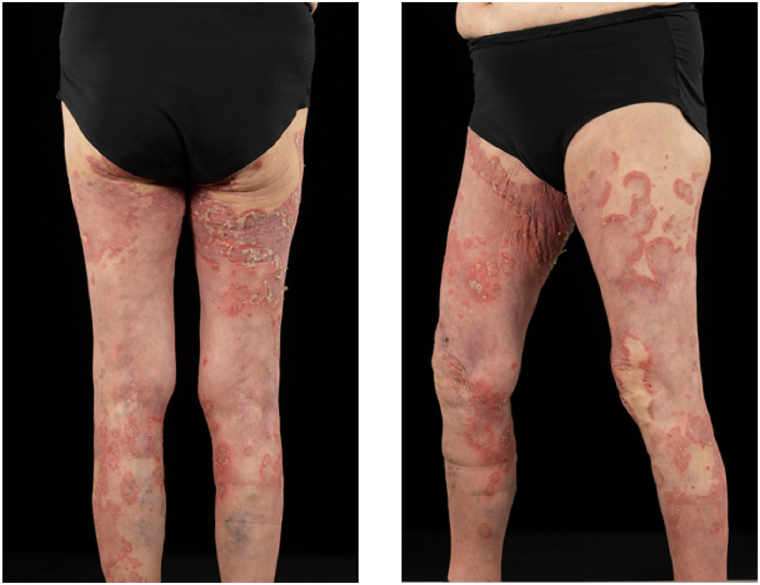
Posterior view of the legs showing a well-demarcated, polycyclic, annular, scaly papulosquamous eruption involving the buttocks and thighs (left panel). The lateral view of the same eruption illustrates the serpiginous and erythematous nature of the plaques (right panel).

A computed tomography scan of the thorax, abdomen, and pelvis ruled out evidence of malignancy. Magnetic resonance imaging of the brain showed chronic alcohol-related cerebellar degenerative changes, and her initial routine laboratory tests, including complete blood count, and renal and liver function tests, were unremarkable apart from mild hyponatremia (131 mmol/L, normal range 135-145 mmol/L) and lymphopenia (1.4 × 10^9^/L, normal range 1.5-4 × 10^9^/L). Skin scrapings were negative for fungal elements. Skin biopsies demonstrated spongiotic dermatitis and hyperkeratosis, parakeratosis with scale crust, and a perivascular inflammatory infiltrate in the papillary dermis (Fig 2, *A,B*). Direct immunofluorescence studies were negative.

**Fig 2:**
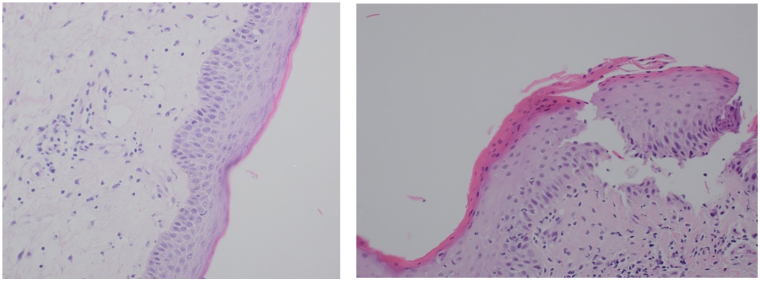
Initial skin biopsy from the right thigh showing hyperkeratosis, acanthosis, and spongiosis with a predominantly lymphocytic infiltrate in the dermis (H&E) (left panel). The second biopsy from the left thigh demonstrated parakeratosis with scale crust and a superficial perivascular lymphocytic infiltrate (H&E) (right panel). *H&E*, Hematoxylin and eosin stain.

**Question 1: Which of the following investigations would help with the final diagnosis?**

**A.** Anti-Ro/SSA antibody
**B.** Serum glucagon levels
**C.** Serum zinc levels
**D.** Antitissue transglutaminase antibodies
**E.** Antinuclear antibodies

**Answer discussion:**

**C.** Serum zinc levels. Acrodermatitis enteropathica is a rare zinc deficiency disorder that may be congenital (due to an autosomal recessive mutation affecting zinc absorption) or acquired (due to nutritional deficiency). The acquired form arises from inadequate zinc intake or impaired absorption, and although more common in malnourished populations, it remains underrecognized in elderly patients with alcohol use disorders.^1^^,^^2^ In this case, the rash was initially misdiagnosed as psoriasis, but failed to respond to systemic and topical therapy. Notably, histopathology did not show classic psoriasiform features — a key clue that should prompt reconsideration of the diagnosis. Our case expands this spectrum by demonstrating how chronic alcohol use can lead to severe zinc deficiency presenting as a psoriasis mimic. Clinicians should maintain a high index of suspicion for nutritional dermatoses when confronted with atypical morphology, poor treatment response, and systemic risk factors.

Zinc plays a vital role in maintaining the epidermal barrier by regulating lipid biosynthesis. It also contributes to cell growth and repair, wound healing, immune response, and antioxidant defense. Treatment with zinc replacement results in clinical improvement within weeks.^3^ Serum or plasma zinc levels and zinc-dependent enzyme levels should be monitored every 3 or 6 months.

## Conflicts of interest

None disclosed.

## References

[1] Osmani S., Smidt A.C., Phan C.M., Johnson D.W. (2021). Acquired acrodermatitis enteropathica from a ketogenic diet. JAAD Case Rep.

[2] Butera A., Agostini M., Cassandri M. (2023). ZFP750 affects the cutaneous barrier through regulating lipid metabolism. Sci Adv.

[3] Park C.H., Lee M.J., Kim H.J., Lee G., Park J.W., Cinn Y.W. (2010). Congenital zinc deficiency from mutations of the SLC39A4 gene as the genetic background of acrodermatitis enteropathica. J Korean Med Sci.

